# Perturbations of Respiratory Rhythm and Pattern by Disrupting Synaptic Inhibition within Pre-Bötzinger and Bötzinger Complexes^1^,^2^,^3^

**DOI:** 10.1523/ENEURO.0011-16.2016

**Published:** 2016-05-13

**Authors:** Vitaliy Marchenko, Hidehiko Koizumi, Bryan Mosher, Naohiro Koshiya, Mohammad F. Tariq, Tatiana G. Bezdudnaya, Ruli Zhang, Yaroslav I. Molkov, Ilya A. Rybak, Jeffrey C. Smith

**Affiliations:** 1 Department of Neurobiology and Anatomy, Drexel University College of Medicine, Philadelphia, Pennsylvania 19129; 2 Cellular and Systems Neurobiology Section, National Institute of Neurological Disorders and Stroke, National Institutes of Health, Bethesda, Maryland 20892; 3 Department of Mathematics and Statistics, Georgia State University, Atlanta, Georgia 30302

**Keywords:** Bötzinger complex, brainstem, central pattern generation, pre-Bötzinger complex, respiration, synaptic inhibition

## Abstract

The pre-Bötzinger (pre-BötC) and Bötzinger (BötC) complexes are the brainstem compartments containing interneurons considered to be critically involved in generating respiratory rhythm and motor pattern in mammals. Current models postulate that both generation of the rhythm and coordination of the inspiratory-expiratory pattern involve inhibitory synaptic interactions within and between these regions. Both regions contain glycinergic and GABAergic neurons, and rhythmically active neurons in these regions receive appropriately coordinated phasic inhibition necessary for generation of the normal three-phase respiratory pattern. However, recent experiments attempting to disrupt glycinergic and GABAergic postsynaptic inhibition in the pre-BötC and BötC in adult rats *in vivo* have questioned the critical role of synaptic inhibition in these regions, as well as the importance of the BötC, which contradicts previous physiological and pharmacological studies. To further evaluate the roles of synaptic inhibition and the BötC, we bilaterally microinjected the GABA_A_ receptor antagonist gabazine and glycinergic receptor antagonist strychnine into the pre-BötC or BötC in anesthetized adult rats *in vivo* and in perfused *in situ* brainstem–spinal cord preparations from juvenile rats. Muscimol was microinjected to suppress neuronal activity in the pre-BötC or BötC. In both preparations, disrupting inhibition within pre-BötC or BötC caused major site-specific perturbations of the rhythm and disrupted the three-phase motor pattern, in some experiments terminating rhythmic motor output. Suppressing BötC activity also potently disturbed the rhythm and motor pattern. We conclude that inhibitory circuit interactions within and between the pre-BötC and BötC critically regulate rhythmogenesis and are required for normal respiratory motor pattern generation.

## Abstract (precis)

The pre-Bötzinger (pre-BötC) and Bötzinger (BötC) complexes are the brainstem compartments containing interneurons considered to be critically involved in generating respiratory rhythm and motor pattern in mammals.

## Visual Abstract

**Figure:**
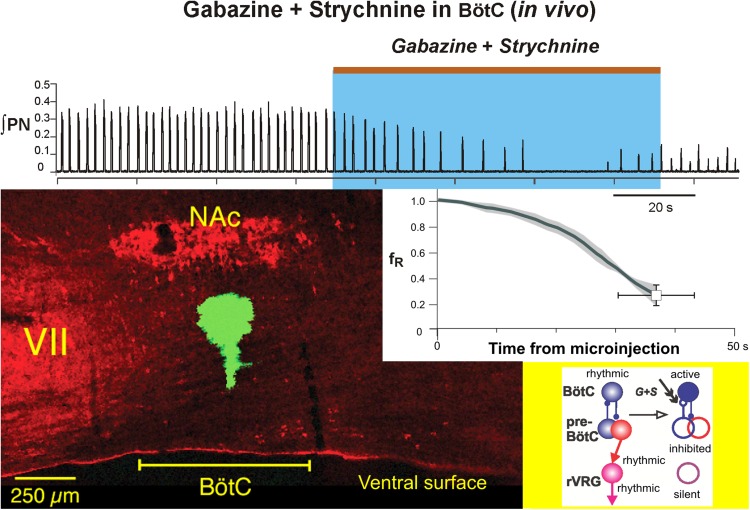

## Significance Statement

Defining functional roles of postsynaptic inhibition in respiratory and other mammalian central pattern generation circuits is a longstanding problem. Inhibitory circuit interactions within and between the brainstem respiratory pre-BötC and BötC have been proposed to be critically involved in normal rhythm and motor pattern generation. A fundamental role of postsynaptic inhibition in these regions has been questioned in recent experiments attempting to pharmacologically disrupt this inhibition. To resolve this contradiction, we applied similar approaches of microinjecting selective pharmacological antagonists of GABA_A_ergic and glycinergic receptor-mediated inhibition in the pre-BötC and BötC in rats. Our results demonstrate large, site-specific perturbations of respiratory rhythm and motor pattern including disruption of rhythmic motor output and thus confirm the critical role of inhibitory circuit interactions.

## Introduction

Rhythmic movements such as breathing and locomotion are produced by central pattern generator (CPG) networks containing interacting excitatory and inhibitory circuits. These circuits are the neural substrates for producing motor behavior (Grillner, 2006) and defining their specific roles in rhythmic motor pattern generation is key to understanding the functional operation of CPGs. Here we have addressed the longstanding problem of defining roles of inhibitory circuits in core structures of the mammalian brainstem respiratory CPG.

The respiratory neural pattern under normal conditions includes three phases: inspiration, post-inspiration, and late expiration (Richter, 1996; Richter and Smith, 2014). The kernel of the respiratory CPG located in the ventral respiratory column of the medulla includes two key compartments, the pre-Bötzinger (pre-BötC) and Bötzinger (BötC) complexes (Alheid and McCrimmon, 2008; Smith et al., 2009, 2013). The pre-BötC contains a heterogeneous population of excitatory neurons, including cells with intrinsic bursting properties, and excitatory synaptic interconnections that generate rhythmic inspiratory activity and drive inspiratory motor output. This excitatory population, when isolated in slices *in vitro*, generates inspiratory bursting activity (Smith et al., 1991; Koshiya and Smith, 1999) that persists after disrupting synaptic inhibition (Johnson et al., 2001). In addition, the pre-BötC contains GABAergic and glycinergic neuron populations (Kuwana et al., 2006; Winter et al., 2009; Morgado-Valle et al., 2010; Koizumi et al., 2013) providing phasic inspiratory inhibition widely distributed in the brainstem including inhibition of BötC expiratory neurons. In turn, BötC post-inspiratory and expiratory neurons provide phasic expiratory inhibition (Jiang and Lipski, 1990; Tian et al., 1999a,b; Ezure et al., 2003a,b) including to pre-BötC inspiratory neurons to coordinate generation of expiratory and inspiratory phases.

The specific contributions of the intrinsic excitatory bursting mechanisms in the pre-BötC, and inhibitory network interactions between pre-BötC and BötC, to respiratory rhythm and motor pattern generation are not clearly understood and this issue is continuously debated. Early theoretical models, based entirely on network inhibitory interactions, could not explain the maintenance of rhythm after blockade of synaptic inhibition *in vitro*. Alternatively, the pure autorhythmic excitatory network models, developed to explain the *in vitro* data, could not account for many behaviors observed *in vivo*, such as the Hering–Breuer inspiratory inhibitory and other respiratory reflexes and the coordinated generation of multiple respiratory phases. Also, these models could not reproduce apneusis, a breathing pattern characterized by a significantly prolonged inspiration alternating with short expiratory intervals (Lindsey et al., 2012).

To resolve this problem, more complicated models have been developed (Rybak et al., 2004, 2007; Smith et al., 2007, 2009, 2013) hypothesizing that: (1) the pre-BötC, although capable of autonomous generation of rhythmic bursting when isolated *in vitro*, is embedded in the larger respiratory network where its activity is controlled by interactions with other brainstem compartments, including inputs from excitatory RTN/pFRG neurons, and from the inhibitory neuron populations in BötC, and (2) both the intrinsic bursting of pre-BötC inspiratory neurons and inhibitory interactions between the neural populations in pre-BötC and BötC are fundamentally involved in generating the normal rhythmic respiratory pattern. Disruption of inhibition in these circuits would lead either to switching to the intrinsic rhythmic activity originating within the pre-BötC, or to sustained or apneustic-like activity.

This concept was challenged by a recent study in the anesthetized rat using targeted pharmacological blockade of fast inhibitory neurotransmission (Janczewski et al., 2013) from which it was concluded that: (1) the BötC does not play a role in respiratory rhythm/pattern generation, and (2) inhibition within the pre-BötC and BötC is not required for generating a normal breathing rhythm and pattern.

The present study was focused on resolving this contradiction and further evaluating roles of inhibitory interactions in pre-BötC and BötC in rhythm generation and shaping respiratory pattern. Our experiments were performed using two distinct preparations, the anesthetized, vagotomized adult rat, and the arterially perfused *in situ* brainstem–spinal cord preparation of juvenile rat. Specific pharmacological blockers of glycinergic (strychnine) and GABA_A_ergic (gabazine) receptor-mediated inhibition were selectively microinjected into the pre-BötC or BötC and perturbations of the respiratory frequency and phases were evaluated. In addition, microinjections of the GABA_A_ receptor agonist muscimol were used to inhibit neural activity in each compartment. Our results were not consistent with those reported by Janczewski et al. (2013). Disrupting inhibition within pre-BötC or BötC, as well as inhibiting BötC activity, caused major perturbations of the respiratory frequency and three-phase pattern. Moreover, blocking inhibition within the BötC lead to apnea confirming the critical role of inhibitory interactions between pre-BötC and BötC. Our results are consistent with previous proposals of the important role of inhibitory interactions within and between pre-BötC and BötC in generating and shaping the respiratory pattern.

## Materials and Methods

### Animal procedures

**Figure 1.**
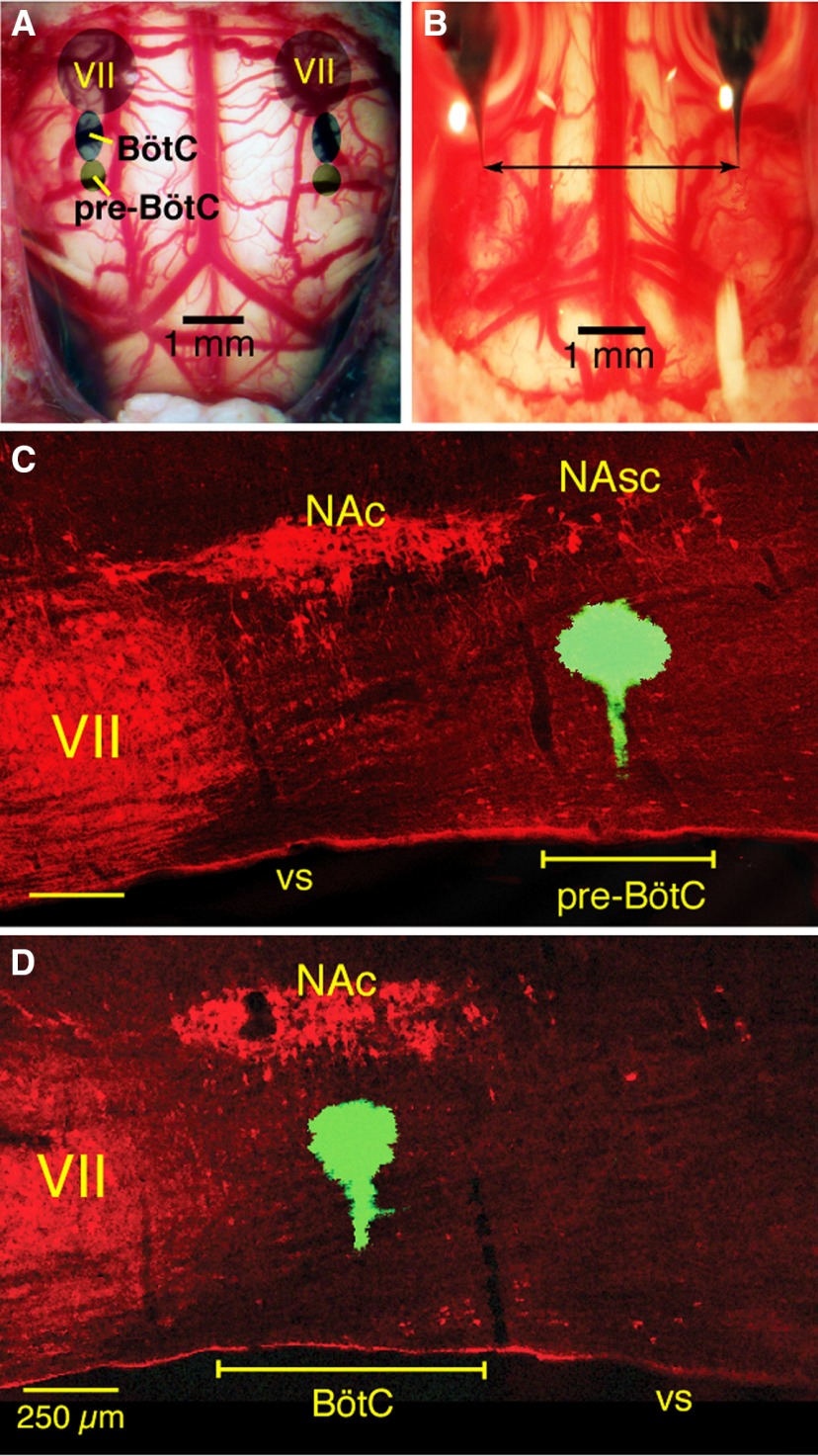
Ventral view of the adult rat medulla and histology illustrating targeted sites for pharmacology experiments *in vivo*. ***A***, Photograph of adult rat brainstem ventral surface as exposed in the *in vivo* experimental preparations with an overview of targeted locations (caudal to facial motor nucleus, VII) of BötC and pre-BötC as routinely identified by electrophysiological mapping of neuronal activity profiles in the present experiments. ***B***, Photomicrograph of ventral medullary surface and bilaterally arranged pipettes (blue dye-filled for visualization) as typically configured for near perpendicular penetrations of the ventral surface for simultaneous microinjections. ***C***, ***D***, Confocal microscopic images of parasagittal histologic sections (50 µm thick) showing, respectively, examples of targeted sites for microinjection of inhibitory antagonists in the pre-BötC ventral to the semicompact subdivision of nucleus ambiguous (NAsc), and in the BötC ventral to the compact subdivision of nucleus ambiguus (NAc). Targeted sites are marked by microinjected solution of fluorescent microspheres (green). NA and VII motoneurons are immunolabeled by ChAT antibody (red). Rostrocaudal spatial extent of the pre-BötC and BotC compartments are indicated. vs, ventral surface.

All experimental procedures used in this study were approved by either the NINDS Animal Care and Use Committee, or the Drexel University Institutional Animal Care and Use Committee, which oversees Drexel University's AAALAC International-accredited animal program. All electrophysiological recording and pharmacological microinjection experiments were performed via a surgically exposed ventral brainstem for access to the ventrolateral medullary pre-BötC and BötC regions (**Fig. 1**).

### Surgical procedures in adult rats *in vivo*

Spontaneously breathing, adult male Sprague-Dawley rats (340–380 g) were anesthetized with isoflurane vaporized in O_2_ (Matrix; 4–5% induction, 1.75–2.0% maintenance) via a snout mask. Anesthetic depth was maintained at a level at which withdrawal reflexes, as well as changes in heart rate and blood pressure in response to pinching the distal hind limbs, were absent. After tracheotomy with a glass tube, animals were artificially ventilated with the same gas mixture (60 min^−1^, 2.5–3.0 ml tidal volume; Columbus Apparatus rodent ventilator). Electrocardiogram was measured via three small subcutaneous electrodes using conventional amplification and filtering (Neurolog; Digitimer) and monitored using an audio amplifier (model AM10; Grass Instruments) and oscilloscope (Tektronix). One femoral artery and vein were cannulated for measurement of arterial pressure and infusion of drugs/saline, respectively. During all surgical procedures, rectal temperature was maintained at 37.0 ± 0.1°C via a servo-controlled heating blanket coupled to a rectal thermometer (Harvard Apparatus). The phrenic nerve (PN) was prepared for recording by dissecting the nerves free from the surrounding tissue. Ventral neck muscles (cleidomastoideus, sternomastoideus, sternohyoideus, omohypides, and digastricus), infrathyroid portions of the trachea and esophagus were removed. The body of the 1st neck vertebra (atlas) and base portion of occipital bone were removed to expose the ventral medulla and the axo-occipital membrane was cut. The dura was then opened using iridectomy scissors and residual bleeding from lateral epidural sinus was arrested by applying small pieces of gelfoam (USP, Pharmacia) soaked with thrombin solution (50 U · ml^−1^ USP, Biopharm Laboratories) dissolved in artificial CSF (aCSF).

All animals were vagotomized and baro- and chemoreceptor denervated via bilateral transection of the carotid sinus nerves to prevent cardiorespiratory reflex influences on motor nerve outputs (Richter and Seller, 1975; Grundy et al., 1986; Hopp and Seagard, 1998; Virkki et al., 2007; Baekey et al., 2010). A bilateral pneumothorax was performed before electrophysiological recording to eliminate lung inflation-related movement artifacts and chest wall mechanoreceptor feedback. A positive end-expiratory pressure of 1.0 cm H_2_O was maintained to prevent lung atelectasis during expiration. Animals were paralyzed by an intravenous bolus injection (2 mg/kg), followed by continuous infusion (3–4 mg/kg/h), of vecuronium bromide (Abbott Laboratories) dissolved in Ringer–Locke solution. End-tidal CO_2_ was maintained between 5.0% and 5.5% (Capstar, CWE) by adjusting frequency of ventilation. If necessary, animals were continuously infused with Ringer–Locke solution (1.0–1.25% body weight or 10.0–12.5 ml/kg/h) to maintain a stable mean arterial pressure of 85–95 mmHg.

### *In situ* arterially perfused brainstem–spinal cord preparation

Experiments were also performed with the *in situ* arterially perfused brainstem–spinal cord preparations from juvenile rats (3–5 weeks old) as previously described (Paton, 1996; Smith et al., 2007). These preparations were studied because they provide the opportunity to investigate roles of synaptic inhibition in an un-anesthetized preparation generating the three-phase respiratory pattern that could be clearly identified from simultaneous recordings of spinal and cranial nerves, including prominent post-inspiratory (post-I) discharge recorded from the central vagus nerve (cVN). We note that cVN recordings from the normocapnic, vagotomized, carotid body denervated, and isoflurane anesthetized adult rats *in vivo* in our experiments do not routinely exhibit post-I discharge although neuronal recording in the BötC always shows post-I activity (see Fig. 3*B*), which is an important feature of medullary respiratory circuit activity. Furthermore, a number of theoretical models (Rybak et al., 2007; Smith et al., 2007; Rubin et al., 2009; Shevtsova et al., 2011, 2014; Richter and Smith, 2014) postulating roles of pre-BötC and BötC inhibitory circuits in respiratory rhythm and pattern generation have been based in part on experimental results obtained from these *in situ* preparations. It is therefore critical to test their roles in this preparation, and to compare results from the *in vivo* anesthetized adult rat preparations using a similar strategy for targeted disruption of synaptic inhibition in the pre-BötC and BötC.

Preheparinized (1000 units, given intraperitoneally) juvenile rats (Sprague-Dawley, 45–90 g; male) were anaesthetized deeply with 5% isoflurane and the portion of the body caudal to the diaphragm was removed. The head and thorax were immersed in ice-chilled carbogenated aCSF solution (in mm: 1.25 MgSO_4,_ 1.25 KH_2_PO_4_, 5.0 KCl, 25 NaHCO_3_, 125 NaCl, 2.5 CaCl_2_, 10 dextrose, 0.1785 polyethylene glycol) and the rat was decerebrated at a precollicular level. The descending aorta, PN, and cVN were surgically isolated. As with the *in vivo* preparations, ventral neck muscles, infrathyroid portions of the trachea, the esophagus, and then the 1st neck vertebra and base portion of the occipital bone were removed to expose the ventral medulla. The axo-occipital membrane was cut, and the dura was then cut open. The preparation was transferred to a recording chamber and secured in a stereotaxic head frame ventral side up. The descending aorta was cannulated with a double-lumen catheter for perfusion and recording of perfusion pressure with a pressure transducer (Micron Instruments). Vecuronium bromide was added to the perfusate to block neuromuscular transmission (4 μg/ml; SUN Pharmaceutical Industries). The perfusate was gassed with 95% O_2_/5% CO_2_ and maintained at 31°C. Vasopressin (200–400 pm as required; APP Pharmaceuticals) was added to the perfusate to raise and maintain perfusion pressure between 70 and 80 mmHg (Paton et al., 2006). Unless stated, all chemicals were from Sigma-Aldrich.

### Electrophysiological recording *in vivo*

With the rat in the supine position, the central ends of the cut PN were placed on bipolar silver hook electrodes for recording (10–5000 Hz bandpass; Neurolog, Digitimer) and immersed in a mineral oil pool formed by skin flaps. To identify precise locations of the BötC and pre-BötC the activity of medullary expiratory and (pre)inspiratory neurons was recorded (200–3000 Hz bandpass; Neurolog) by a ventral approach and glass (WPI) microelectrodes (tip outer diameter of 2–3 µm, 5–10 MΩ) filled with 0.5 m NaCl and 2% pontamine sky blue. The pre-BötC is readily identified by a characteristic pattern of pre-inspiratory/inspiratory (pre-I/I) activity (see **Fig. 3*A***) and the BötC has a characteristic profile of post-I and augmenting expiratory (aug-E) activities (see **Fig. 3*B***). The microelectrode was held in a three-dimensional stepper motor assembly (DC-3K, Märzhäuser), attached to the rail of the stereotaxic frame, and advanced in steps of 2.5–5.0 µm. After mapping neuronal activity, the medullary surface was marked bilaterally by iontophoresis for 15 min of the pontamine sky blue dye (−25 nA, 0.125 Hz, 4 s; Axoclamp 2A) as a guide spot for insertion of drug microinjection pipettes. All electrophysiological signals were recorded simultaneously with expiratory CO_2_ level, arterial blood pressure, and lung inflation pressure, on the hard disk of a personal computer via a 16-bit analog-to-digital converter (PowerLab, AD Instruments, 10 kHz sampling rate) and displayed continuously with software (Chart, AD Instruments).

### Electrophysiological recording *in situ*

To monitor respiratory network activity and motor output in the *in situ* perfused brainstem–spinal cord preparations, we recorded with fire-polished glass suction electrodes inspiratory activity from PN, and cVN inspiratory and post-inspiratory activity. Signals were amplified (50,000–100,000×; CyberAmp 380, Molecular Devices), band-pass filtered (0.3–2 kHz), digitized (10 kHz sampling rate) with an AD converter [Cambridge Electronics Design (CED)], and then rectified and integrated digitally with Spike 2 software (CED). Extracellular population activity from pre-BötC or BötC respiratory neurons in the perfused *in situ* preparations was also recorded with a fine glass electrode (3–5 MΩ) filled with 0.5 m Na^+^ acetate.

### Targeting the pre-BötC and BötC regions with microinjections *in vivo*

**Figure 2.**
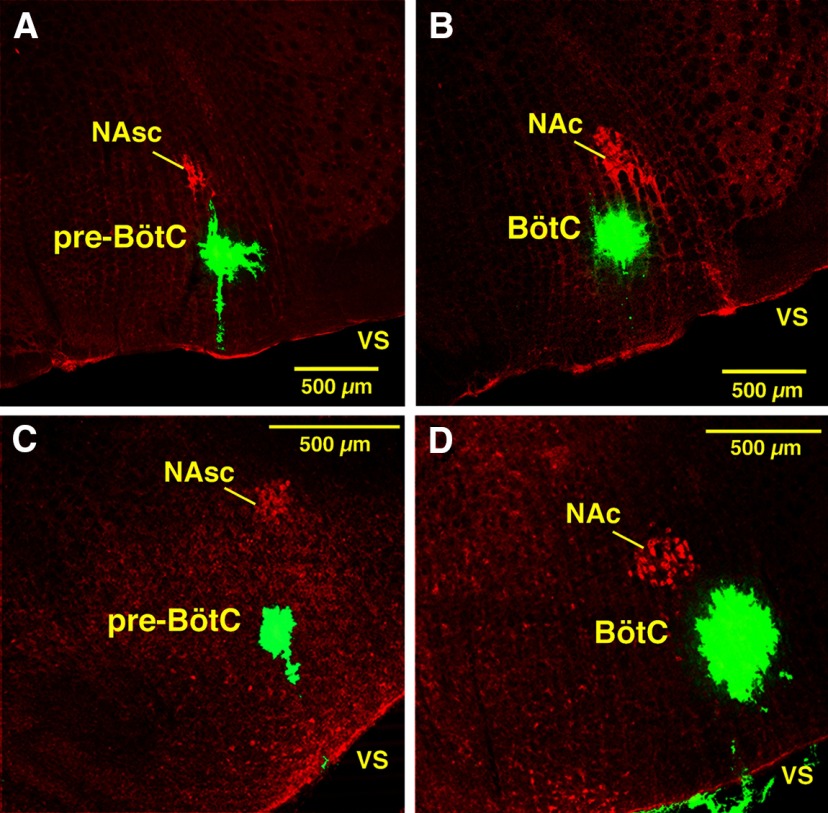
Confocal microscopic images of histologic sections illustrating *post hoc* validation of microinjection sites marked by fluorescent microspheres (green) in the pre-BötC or BötC regions in fixed sections from anesthetized adult rat *in vivo* preparations and juvenile rat *in situ* brainstem–spinal cord preparations. ***A***, ***B***, Coronal sections (30 µm thick) of fixed tissue at the level of pre-BötC (***A***) and BötC (***B***) from *in vivo* preparations. ***C*, *D*,** Coronal sections (30 µm) of fixed tissue at pre-BötC (***C***) and BötC (***D***) levels from juvenile rat *in situ* brainstem–spinal cord preparations. Subdivisions of nucleus ambiguus (NAc, compact subdivision; NAsc, semi-compact subdivision), labeled with ChAT antibody (red), provide regional landmarks for pre-BötC (ventral to NAsc) and BötC (ventral to NAc) levels of the medulla. Each image is taken from serial coronal histologic sections obtained from experiments targeting these regions. Labeling with microinjected solution of fluorescent microbeads, with varying extent of local spread, indicates the approximate center of the drug microinjection sites in these examples. vs, ventral surface.

**Figure 3.**
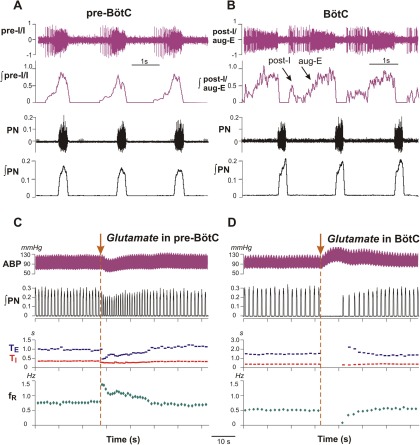
Characteristic profiles of extracellularly recorded neuronal population activity and examples of perturbations of inspiratory motor output activity and blood pressure produced by pharmacological excitation of neurons within the pre-BötC or BötC regions in the adult rat *in vivo*. **A**, A typical example of pre-inspiratory/inspiratory (pre-I/I) population activity used for the identification of pre-BötC. **B**, An example of post-I and aug-E population activity (post-I/aug-E, simultaneously recorded in this example) used for the identification of BötC. In both ***A*** and ***B***, the raw recording from the phrenic nerve (PN) and PN integrated activity (∫PN**)** are shown at the bottom. ***C***, A 500 ms duration microinjection of L-Glu (Glutamate) in the pre-BötC produced an increase in the ∫PN burst frequency (see the trace for integrated PN activity, ∫PN), and a transient decrease in the ABP. The respiratory frequency (*f*_R_; bottom trace, green) increased primarily due to reduction in expiratory phase duration (T_E_ trace, blue) at a relatively unchanged inspiratory duration (T_I_ trace, red). ***D***, A microinjection of L-Glu in the BötC caused a rapid suppression of PN activity (see traces for ∫PN and inspiratory, T_I_, and expiratory, T_E_, durations) accompanied by an increase of ABP. In ***C***and ***D***, L-Glu (10 mm, 5 nl) was microinjected bilaterally during the brief (500 ms) pulse; the moments of injections are indicated by brown arrows. Traces for T_I_, T_E_, and *f*_R_, represent corresponding running time intervals of these parameters before, during, and recovery from L-Glu microinjection.

To block fast inhibitory transmission a cocktail of GABA_A_ (gabazine, Sigma-Aldrich) and glycine (strychnine, Sigma-Aldrich) receptor antagonists (in aCSF) was pressure injected (250 µm, 105–115 nl) into the BötC or pre-BötC bilaterally and simultaneously (1–1.5 nl/s) with small diameter (OD = 15 µm, ID = 7.5 µm) polished micropipettes (Drummond Scientific). In another set of experiments, the GABA_A_ agonist muscimol was injected (100 µm, 25–30 nl) into the BötC or pre-BötC bilaterally and simultaneously. Control injections were made at the same sites with aCSF alone. All injection volumes were measured by microscopically observing the change in level of the meniscus within the micropipette. Microinjections were done after electrophysiological recording to map the extracellular single unit/neuronal population activities characteristic of the pre-BötC or BötC regions as described above, and also in some *in vivo* experiments after pharmacological probing by microinjection of 5 nl of 10 mm l-glutamate (l-Glu) to further confirm locations of the pre-BötC or BötC regions, which have site-specific responses to brief (500 ms) local l-Glu microinjections (i.e., transient apnea with increasing arterial blood pressure (ABP) for the BötC region, and tachypnea with decreasing ABP for the pre-BötC region; see **Fig. 3*C*,*D***). These blood pressure responses are typical because the pre-BötC partially overlaps with the depressor caudal ventrolateral medulla and the BötC with the pressor rostral ventrolateral medulla (RVLM; Kanjhan et al., 1995; Lipski et al., 1996; Moraes et al., 2012). According to our mapping by extracellular recordings, and consistent with characteristic perturbations obtained by our targeted l-Glu microinjections based on the neuronal activity maps, BötC and pre-BötC in isoflurane anesthetized adult male rats (340–380 g) occupy restricted areas: 1.8–2.1 mm lateral, 550–850 µm depth from the ventral surface, and extending ∼400 µm in the rostrocaudal dimension (i.e., ∼800–1200 µm from the caudal pole of facial nucleus or ∼1.6–2.0 mm rostral to obex) for pre-BötC, and for BötC 1.9–2.2 mm lateral, 450–750 µm depth, and 600–700 µm in the rostrocaudal dimension (∼100–750 µm from the caudal pole of facial nucleus or ∼2.0–2.75 mm rostral to obex). At the end of each experiment fluorescent marker yellow-green microbeads (0.1 µm diameter, 2% solution, Invitrogen) were injected via a pipette (OD = 30 µm, ID = 15 µm; Drummond) into the site of microinjections for *post hoc* morphological verification (**Figs. 1**, **2**).

Because the locations of pre-BötC and BötC in adult rats relative to the ventral surface are shallow (550–850 and 450–750 µm, respectively), we performed tests for back leakage of injected solution along the microinjection pipette track. Pipettes with different outer diameters (12.5–50 µm) were filled with 2% of pontamine sky blue and only large diameter pipettes (>30 µm) showed substantial leak of dye back to the ventral surface during microinjections. Accordingly, for drug microinjections *in vivo* we only used pipettes with 15 µm OD that never exhibited back leak.

### Microinjections in arterially perfused brainstem–spinal cord preparations *in situ*

As *in vivo*, we bilaterally microinjected a cocktail of gabazine and strychnine and in some experiments muscimol (all drugs dissolved in the perfusate aCSF) with micropipettes (ID = 20 µm) positioned in the pre-BötC or BötC through the ventral medullary surface with microdrives (Marzhauser) after mapping locations of these regions with extracellular recording of pre-inspiratory (pre-BötC) or expiratory (BötC) neuronal population activities. Slow, continuous microinjection was accomplished by applying low pressure (30 mm Hg) to the pipettes with a pressure control and measurement system. In preliminary experiments we determined that, compared with the *in vivo* experiments, lower concentrations of drugs (30 µm of gabazine and strychnine or 10 µm muscimol) bilaterally injected into either of these regions caused rapid and large disturbances of respiratory motor output and hence all experiments with the perfused brainstem–spinal cord preparations were conducted at these lower drug concentrations. In some experiments, extracellular recordings of neuronal population activity in pre-BötC (during injections in BötC) or BötC (during injections in pre-BötC) regions were made during drug microinjections. Fluorescent microbeads were microinjected to mark the sites of drug microinjection (**Figs. 1, 2**) as described above for the *in vivo* experiments.

### Histologic verification of microinjection sites

Immediately after marking locations of drug microinjection sites, animals *in vivo* were transcardially perfused with 400 ml of saline (10–12°C, pH 7.4) with heparin (1000 U/ml) followed by 500 ml of 4% paraformaldehyde (wt/vol) in 0.1 m PBS (10–12°C, pH 7.4). A similar procedure was followed (but with smaller volumes of saline and paraformaldehyde perfusates) for perfusion fixation of the *in situ* brainstem–spinal cord preparations. For both preparations, the brainstem was removed, postfixed in the same fixative for 24 h at 4°C, and subsequently cryoprotected by sequential incubation in 15% and 30% sucrose in PBS and stored overnight at 4°C in 30% sucrose, 0.1 m PBS solution. The medulla oblongata was sectioned parasagittaly or coronally at 30- or 50-µm-thick sections with a freezing microtome. For fluorescence immunohistochemistry, floating sections were incubated with 10% donkey serum in PBS with Triton X-100 (0.3%) and subsequently incubated for 48–72 h at room temperature with primary antibodies for choline acetyltransferase (ChAT; goat anti-ChAT, Millipore, 1:200) to label motoneurons. Sections were then rinsed with PBS and incubated for 2 h with secondary antibodies for ChAT (donkey anti-goat-Dylight 488, 1:500). Individual sections were mounted on slides and covered with an anti-fading medium (Fluoro-Gel; Electron Microscopy Sciences). Fluorescent labeling was visualized with a laser-scanning confocal imaging system (Zeiss LSM 510).

### Signal analyses of respiratory parameters

All automated analyses of respiratory parameters from digitized nerve or neuronal population activities were performed with IDL software (Exelis VIS). Inspiratory events were detected from digitally rectified and integrated phrenic nerve signals via a 200 ms window moving average and peak detection algorithm that calculated a threshold-based zero derivative (positive peak) point. Following peak detection, interburst interval (IBI; inverse of burst frequency), inspiratory time (T_I_) and expiratory time (T_E_) were measured. T_I_ was measured as the original integrated burst width at 20% of the peak height above baseline; T_E_ was calculated as IBI – T_I_. Inspiratory amplitude (amp) was calculated by subtracting the local baseline from the peak value. The endpoint of the parameter quantification was defined when the perturbation during or after microinjections reached it maximum or the signals declined to noise level and the program started missing peak detections (which could appear as a quantum jump in IBI).

**Figure 4.**
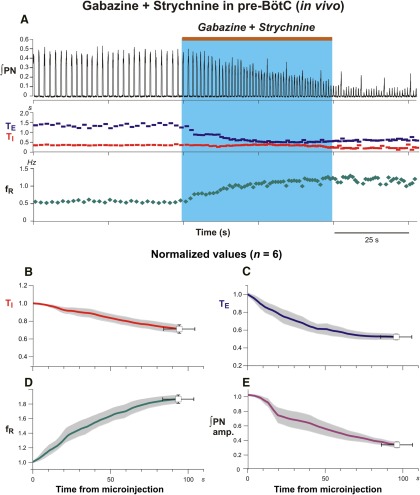
Perturbations of respiratory activity by pharmacologically disrupting GABA_A_ergic and glycinergic inhibition in the pre-BötC of anesthetized adult rat *in vivo*. ***A***, Simultaneous bilateral microinjections of gabazine and strychnine (both 250 µm delivered by slow microinjections of 110 nl during the time interval indicated by the brown bar at the top and blue rectangle) caused an increase of respiratory frequency and a reduction in the integrated phrenic nerve activity amplitude (∫PN; black trace, top). The increase of respiratory frequency (*f*_R_; green trace, bottom) was mainly due to reductions of expiratory phase duration (T_E_, blue trace) accompanied by only a small increase of inspiratory phase duration (T_I_, red trace). ***B***–***E***, Group mean time series showing developing changes of normalized T_I_ (***B***, red curve), T_E_ (***C***, blue curve), *f*_R_ (***D***, green curve), and ∫PN amp (***E***, magenta curve) computed over the time window shown from the start (time = 0) of microinjection. Data were computed from ∫PN for this representative experimental group (*n* = 6). Solid colored curves are group mean time and mean normalized parameter values; gray shaded bands are ±1 SEM for the mean parameter values. Endpoints shown are mean time and normalized parameter values ±1 SEM for both at the maximal perturbation for the injection periods used.

The time courses of the changes in the above parameters for a given experimental group were variable, which we assume resulted from variability in the times required for drug diffusion to affect a sufficient number of neurons to produce the perturbations. To represent group data, we computed the mean time courses of the parameter values by the following procedures. For each experiment, the time courses of the parameters were extracted by a 30 s window moving median up to the defined endpoint, and the parameter values for each time series were normalized to the computed mean values during the control period (from 120 to 0 s before start of microinjection). Each time course was divided into 100 time points representing 1% increments from the start of microinjection (time 0) to the endpoint. We then computed the group mean time, and also mean values of the normalized parameters, at each of these points, which were plotted and connected by lines to represent the mean time series. The mean endpoint was plotted with its ±SEM represented by crossbars (see **Figs. 4*B*–*E*, 6*A*–*D*, 7*B–E*, 9*A*–*D*, 13*B*–*E*, and 14*D*–*G***) and the SEM of the preceding normalized parameter values for the group time series were represented by a gray band. To determine statistical significance, the control values were compared with the endpoint values for each experiment within a group using a two-sided Wilcoxon signed-rank test (significant *p* value < 0.05).

In addition to quantifying the respiratory parameters indicated above, we analyzed perturbations of amplitudes and durations of individual phases of the respiratory cycle (e.g., post-I activity in cVN recordings *in situ*) from integrated, cycle phase-triggered (peri-event) neurograms aligned at the onset of the inspiratory phase defined by PN activity. Successive cycle-triggered traces were either overlaid, or represented as a colored raster plot to depict temporal profiles of activity intensity before and during/after drug injection periods (see **Figs, 10*A*,*B*, 14*B***).

## Results

### Perturbations of respiratory rhythm and pattern by targeted microinjection of l-glutamate in the pre-BötC or BötC *in vivo*

As described in Materials and Methods, we microinjected l-Glu in pre-BötC and BötC regions (*n* = 15, 10 mm, 5 nl) to establish characteristic regional excitatory perturbations. These *in vivo* experiments demonstrate that activation of pre-BötC (*n* = 5) by brief (500 ms) l-Glu microinjections always produced a transient increase of respiratory frequency (from 28.9 ± 4.4 to 44.55 ± 6.1 bursts/min, *p* < 0.0001) due to a significant reduction of expiratory phase duration (T_E_; **Fig. 3*C***). In contrast, the same l-Glu microinjections into BötC (*n* = 10) always transiently prolonged the respiratory period (by 5.4 to 21 s range; mean value = 10.1 ± 4.03 s; **Fig. 3*D***), documenting site-specificity of the perturbations.

### Perturbations of respiratory rhythm and pattern by microinjection of gabazine and strychnine in the pre-BötC *in vivo* and *in situ*

Microinjections of the gabazine-strychnine cocktail were performed after initially identifying the pre-BötC region electrophysiologically by recording pre-I/I neuronal population activity and/or also after microinjected l-Glu (*in vivo*) to identify the pre-BötC region (**Fig. 3*C***). Perturbations of neural activity with bilateral microinjections of 110 nl of 250 µm of gabazine and strychnine in the anesthetized adult rat *in vivo* were stereotypical. A representative example is shown in **Fig. 4*A*.** Drug microinjections in pre-BötC produced an increase in the respiratory frequency (*f*_R_), primarily due to shortening of T_E_, as well as a decrease of the amplitude of integrated PN activity. The group (*n* = 6) *in vivo* data are summarized in **Figures 4*B*–*E***, which shows the mean time courses of the developing perturbations of all parameters (normalized to control values) during and after the microinjections. Perturbations developed rapidly, within 10 s following the onset of microinjection in all cases. The respiratory frequency increased to 184.8 ± 5.6% (*p* = 0.03), T_I_ decreased to 71.3 ± 4.5% (*p* = 0.03), and T_E_ was reduced to 52.6 ± 3.2% (*p* ≤ 0.03) of control values. The amplitude of integrated PN activity decreased to 32.1 ± 3.9% (*p* ≤ 0.03) of pre-injection control values. These parameter values, all of which are statistically significant, were obtained at 95.83 ± 10.03 s after onset of the microinjections when the perturbations approached maximum values. The average injection time for the group was 57.66 ± 22.09 s. After these perturbations, the time required for recovery of inspiratory activity with a pattern resembling the control activity was variable, but usually a period of minimally 40 min was required *in vivo*.

**Figure 5.**
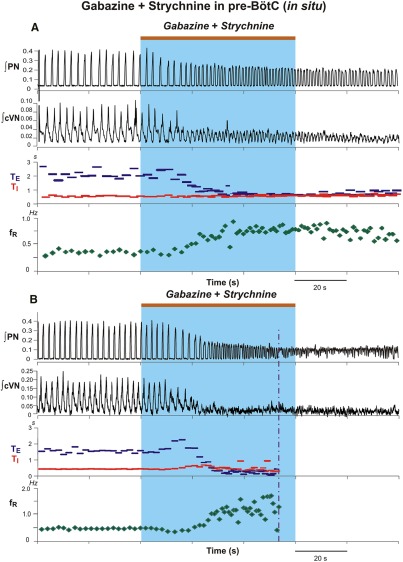
Perturbations of respiratory activity by pharmacologically disrupting GABA_A_ergic and glycinergic inhibition in the pre-BötC of juvenile rat perfused brainstem–spinal cord *in situ*. ***A***, Example of experimental recordings illustrating perturbations of integrated PN (∫PN) and cVN (∫cVN) activities by simultaneous bilateral microinjections of gabazine and strychnine (both 30 µm; injection period indicated by the brown bar at the top and blue rectangle), which caused an increase of respiratory frequency (*f*_R_) due to a reduction in T_E_ (blue) without significant changes in T_I_, and a reduction in amplitude of ∫PN and ∫cVN. ***B***, Example of perturbations where the ∫PN activity progressed to tonic activity, as indicated by the upward shift of the ∫PN signal baseline. Analysis of respiratory parameters in this case was performed up to the time point indicated by the vertical dot-dashed line. The progressive reduction in ∫cVN amplitude in both examples reflects in part a reduction and eventual loss of post-I activity (Fig. 10 shows a more detailed analysis).

**Figure 6.**
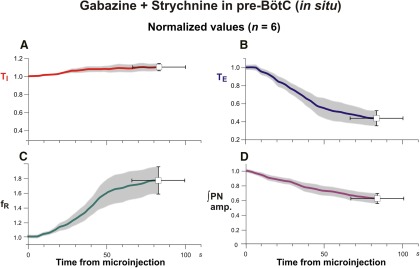
Group data summarizing changes of respiratory activity parameters by pharmacologically disrupting GABA_A_ergic and glycinergic inhibition in the pre-BötC of juvenile rat perfused brainstem–spinal cord *in situ*. ***A*–*D***, Mean time series from the start of microinjection showing developing changes of normalized T_I_ (***A***, red curve), T_E_ (***B***, blue curve), respiratory frequency (*f*_R_; ***C***, green curve), and integrated PN discharge amplitude (***D***, amp, magenta curve), which were computed from integrated phrenic nerve activity for a representative experimental group (*n* = 6). Solid colored curves are group mean time and normalized parameter values; gray bands are ±1 SEM for the mean normalized parameter values as in Figure 4*B*–*E*.

Similarly rapid and large perturbations were caused by bilateral microinjections of 30 µm gabazine-strychnine within the pre-BötC of *in situ* perfused juvenile rat brainstem–spinal cord preparations (*n* = 6; **Fig. 5*A*,*B***). Figure 5*A* shows a typical example of the large disturbances of the respiratory frequency and amplitudes of activity recorded from PN and cVN, including the pronounced increase in the frequency of PN inspiratory discharge accompanied by reductions in discharge amplitude. In the example shown in **Figure 5*B***, the augmented frequency of PN inspiratory discharge culminated in tonic activity, which occurred in one of six experiments. For the group data mean values of respiratory frequency increased to 175.9 ± 18.0% (*p* = 0.03), T_I_ slightly increased to 110.4 ± 4.0% (*p* = 0.03), and T_E_ decreased to 43.7 ± 8.0% (*p* = 0.03) of control values (**Fig. 6*A*–*C***). The integrated PN activity amplitude decreased to 62.3 ± 6.0% (*p* = 0.03; **Fig. 6*D***). These disturbances followed a similar time course to those *in vivo* and were measured at 84.1 ± 17 s after the onset of microinjections (average injection time of 178.3 s) when maximum changes in parameter values obtained. Recovery of control patterns of nerve activity typically occurred 15–20 min after terminating the bilateral microinjections in these *in situ* experiments.

### Disruption of respiratory rhythm and pattern by microinjection of gabazine and strychnine in the BötC *in vivo* and *in situ*

**Figure 7.**
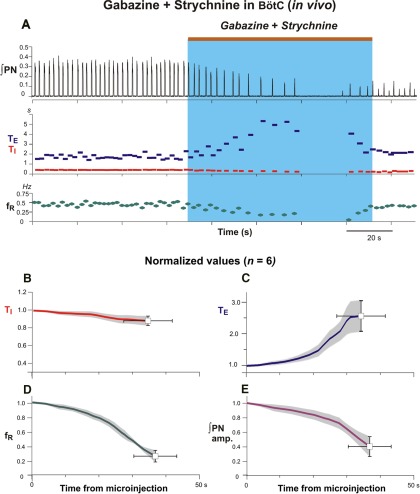
Perturbations of respiratory activity by pharmacologically disrupting of GABA_A_ergic and glycinergic inhibition in the BötC of adult rat *in vivo*. ***A***, Gabazine and strychnine cocktail (110 nl, 250 µm) slowly injected during time period indicated (by the brown bar at the top and blue rectangle), progressively reduced respiratory frequency leading to transient apnea in this example (∫PN, black trace, top). Changes in the inspiratory (T_I_, red) and expiratory (T_E_, blue) phase durations and respiratory frequency (*f*_R_) are shown at the bottom; *f*_R_ was reduced mainly due to prolongation of T_E_. ***B***–***E***, Mean time series from the start of microinjection showing developing changes of normalized T_I_ (***B***, red curve), T_E_ (***C***, blue curve), *f*_R_ (***D***, green curve), and integrated PN activity amplitude (***E***, magenta curve) for a representative experimental group (*n* = 6). Solid colored curves are group mean time and normalized parameter values; gray bands are ±1 SEM for the mean normalized parameter values.

**Figure 8.**
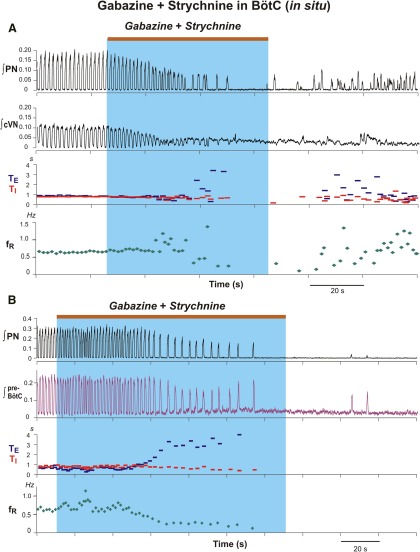
Disruption of rhythmic respiratory activity by block/attenuation of GABA_A_ergic and glycinergic inhibition in the BötC of *in situ* perfused brainstem–spinal cord preparations. ***A***, ***B***, Two examples from experiments using different preparations illustrating perturbations caused by microinjections of gabazine and strychnine (30 µm slowly injected during the period indicated by the brown bars at the top and blue rectangles). As in *the vivo* experiments, respiratory frequency was reduced by a prolonged T_E_, integrated phrenic nerve discharge amplitude (∫PN) was also reduced, and ultimately apnea occurred. Simultaneously recorded integrated cVN (∫cVN) in ***A*** shows disruption of rhythmic activity and tonic discharge (shift of integrated activity baseline) during apneic period. ***B***, Simultaneously recorded integrated pre-BötC pre-I/I population activity from another experiment also reflects the reduction of inspiratory frequency and termination of rhythmic activity during bilateral microinjections of the inhibitory receptor blockers in BötC.

**Figure 9.**
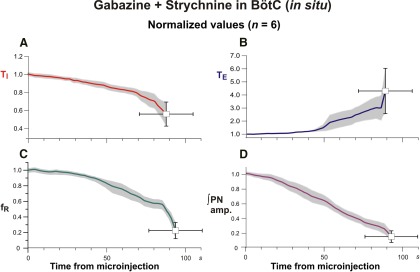
Group data (*n* = 6) summarizing perturbations of respiratory rhythm and motor output pattern parameters by disrupting GABA_A_ergic and glycinergic inhibition in the BötC of *in situ* perfused brainstem–spinal cord preparations. Solid colored curves in these time series are mean time and normalized parameter values and gray bands are ±1 SEM for the normalized values of T_I_ (***A***), T_E_ (***B***), respiratory frequency (*f*_R_; ***C***), and integrated inspiratory activity amplitude (***D***) computed from the start of microinjections from recordings of integrated PN activity.

Bilateral microinjections of gabazine-strychnine were performed in the BötC region after identifying this region by extracellular recording of augmenting expiratory and/or post-I neuronal population activity *in situ* and *in vivo,* and in some (*n* = 3/6) of the *in vivo* experiments in this group, after confirming the characteristic suppression of recorded phrenic discharge and pressor blood pressure responses by l-Glu microinjections. In contrast to the results obtained with microinjections in the pre-BötC, bilateral microinjections of 110 nl of 250 µm gabazine and strychnine into the BötC *in vivo* (**Fig. 7**; *n* = 6), and also the microinjections in the *in situ* experiments with the lower concentrations of the inhibitory receptor antagonists (**Figs. 8, 9**; *n* = 6), significantly reduced the frequency of integrated PN discharge due primarily to a prolongation of T_E_, accompanied by a reduction of T_I_ and the amplitude of integrated PN activity. For the averaged group *in vivo* data (**Fig. 7*B*–*E***), at 36.66 ± 6.4 s following the onset of microinjections, the respiratory frequency decreased to 25.8 ± 8.0% (*p* ≤ 0.03), T_I_ was reduced to 88.4 ± 5.3% (*p* ≤ 0.06), and T_E_ increased to 257.2 ± 48.0% (*p* ≤ 0.03) of control values. The amplitude of integrated phrenic discharge decreased to 39.9 ± 13.8% (*p* ≤ 0.03) of control values (**Fig. 7*E***). In two *in vivo* experiments, rhythmic inspiratory discharge was completely suppressed for ≥20 s (**Fig. 7*A***). The average microinjection time for the group was 53.67 ± 19.32 s.

Similar perturbations occurred with bilateral microinjections of 30 µm gabazine-strychnine in the BötC *in situ* (**Figs. 8**, **9**). For the averaged group data (n = 6, **Fig. 9*A*–*C***) at 92.5 ± 16.77 s after injection onset, inspiratory frequency decreased to 22.6 ± 10.7% (p = 0.03), T_E_ increased to 430.2 ± 17.3% (*p* = 0.03), and T_I_ decreased to 56.2 ± 13.0%, (*p* = 0.03) of pre-injection control values. Integrated PN discharge amplitude was reduced to 15.4 ± 7.0% (*p* = 0.03; **Fig. 9*D***). In three of these *in situ* experiments, rhythmic inspiratory activity was transiently suppressed for ≥20 s (**Fig. 8**). These perturbations were reflected in simultaneously recorded cVN inspiratory activity (**Fig. 8*A***) in all experiments analyzed and by recorded pre-BötC pre-I/I population activity (**Fig. 8*B***) in two of these experiments, which demonstrate that the perturbations in BötC disrupt pre-BötC neuronal activity. In all experiments post-I cVN discharge was also disrupted (below) and in one experiment tonic discharge on the cVN was recorded during the disruption of rhythmic motor output (**Fig. 8*A***). With the bilateral injections of the inhibitory receptor antagonists in the BötC *in situ* or *in vivo*, although nerve activity could emerge after transient suppression of motor output (Figs. 7*A*, 8*A*), such activity was disturbed relative to control activity. In all of these experiments, typically 15–25 min *in situ* and 40–60 min *in vivo* were required to recover activity resembling control patterns of motoneuronal discharge.

### Disruption of three-phase respiratory pattern by blocking inhibition in the pre-BötC or BötC *in situ*

**Figure 10.**
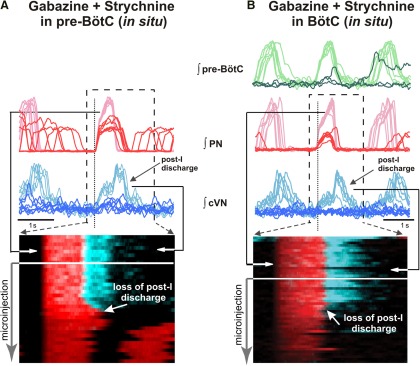
Disturbances of three-phase respiratory pattern including disruption of post-I activity by bilaterally microinjected gabazine and strychnine (30 µm each) in the pre-BötC (***A***) or BötC (***B***) of *in situ* perfused juvenile rat brainstem–spinal cord preparations. Upper traces in ***A*** and ***B*** show seven consecutive overlaid and aligned integrated PN (red traces) and cVN (blue traces) activity signals in the pre-microinjection control period (light red and light blue larger amplitude traces, respectively) and also seven overlaid traces of recorded signals during microinjections (dark red and dark blue traces with reduced amplitudes). Simultaneously recorded pre-BötC pre-I/I population activity before and during microinjections (light and dark green traces, respectively) is also shown at the top in ***B*** to indicate activity perturbations in this region occurring with disruption of inhibition in BötC. Signals are aligned (cycle-triggered) at the onset of PN inspiratory activity indicated by the vertical dotted line in ***A*** and ***B***. Raster plots below overlaid traces show consecutive series of inspiratory-onset aligned respiratory cycles with integrated PN inspiratory (red) and cVN including post-I (blue) activities before and during microinjections. Arrows on the raster plots indicate time interval over which the overlaid traces above the raster plots were obtained for the pre-microinjection period. During microinjections in the pre-BötC or BötC, the amplitude of inspiratory and post-I activity is progressively reduced, and post-I discharge is eliminated as seen on the aligned (darker) ∫cVN traces above the raster plots and in the raster plots with loss of post-I discharge indicated (white arrow).

The microinjections of gabazine-strychnine in either the pre-BötC (*n* = 6) or BötC (*n* = 6) *in situ* disrupted the three-phase respiratory motor output pattern as analyzed from simultaneous recordings of PN and cVN activity, the latter of which always exhibited prominent post-I discharge. Cycle-triggered averages and time-series raster plots of integrated PN and cVN shown in **Figure 10*A*,*B*** illustrate that cVN post-I activity is eliminated as GABA_A_ergic and glycinergic inhibition is attenuated in either region. This loss of post-I activity as the drug-induced perturbation develops represents a transformation from a three-phase to a two-phase motor output pattern (Smith et al., 2007).

### Site-specific perturbations of rhythm and pattern by microinjections of muscimol in the pre-BötC and BötC *in vivo* and *in situ*

**Figure 11.**
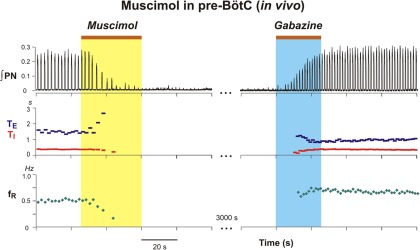
Suppression of rhythmic inspiratory activity by bilateral microinjection of muscimol in the pre-BötC of adult rat *in vivo* and gabazine antagonism of GABA_A_ receptor activation. Muscimol (30 nl, 100 µm) microinjection (during yellow shaded area) rapidly reduced integrated PN activity and produced a long-lasting suppression of inspiratory activity that could be restored by bilateral microinjection of gabazine (110 nl, 250 µm) at the same site in the pre-BötC (blue shaded area). ∫PN, phrenic nerve integrated activity; *f*_R_, respiratory frequency.

**Figure 12.**
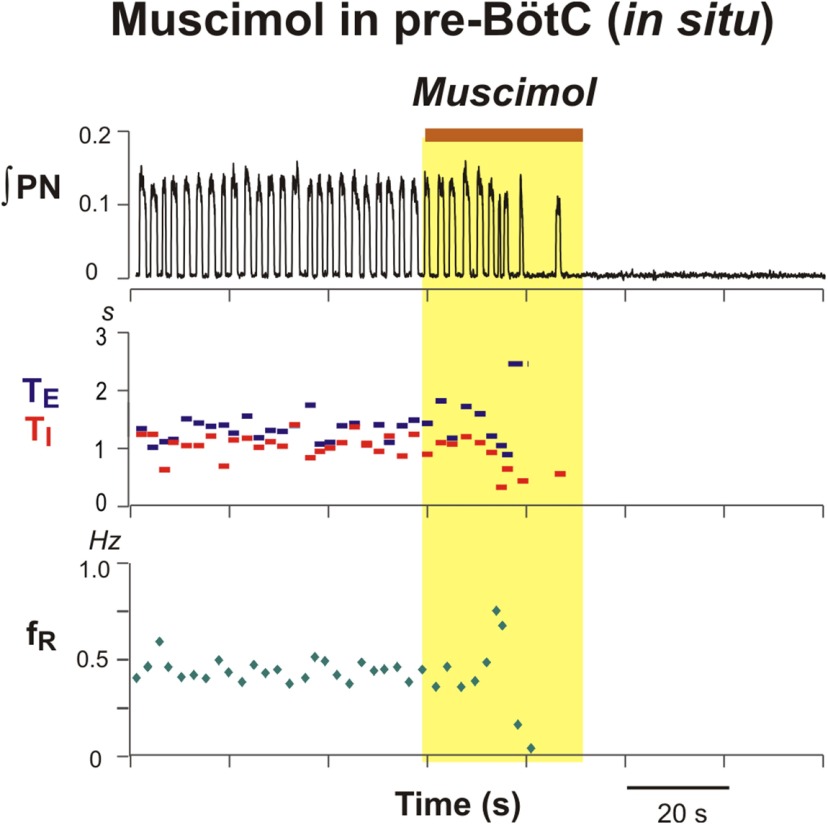
Illustration of prolonged suppression of rhythmic inspiratory activity by bilateral microinjection of muscimol (10 µm) in the pre-BötC (yellow shaded area) of juvenile rat perfused brainstem–spinal cord *in situ*. As *in vivo*, muscimol rapidly reduces phrenic nerve inspiratory discharge frequency and terminates inspiratory activity. ∫PN, phrenic nerve integrated activity; *f*_R_, respiratory frequency.

The differential perturbations of inspiratory discharge frequency with the GABA_A_ and glycine receptor antagonists in the BötC (frequency decrease) versus the pre-BötC (frequency increase) demonstrate site-specificity of the perturbations. To further test for site-specific perturbations by manipulating local inhibition, we regionally inhibited/attenuated neuronal activity by bilateral microinjection of the GABA_A_ receptor agonist muscimol. In these experiments, the pre-BötC and BötC regions were first identified electrophysiologically by extracellular recording and/or by microinjection of l-Glu *in vivo*. As illustrated in **Figure 11**, bilateral microinjection of 25–30 nl of 100 µm muscimol in the pre-BötC *in vivo* progressively reduced the frequency and amplitude of phrenic discharge and eliminated inspiratory motor output within 20 s in all experiments (*n* = 7). The suppression of inspiratory activity persisted for 40.1 ± 26.8 s during which rhythmic inspiratory activity could be restored immediately by subsequent bilateral microinjection of 110 nl of 250 µm gabazine at the same site (**Fig. 11**). This latter result also demonstrated the efficacy of gabazine at the concentrations used in our experiments to antagonize activation of GABA_A_ receptors in a regionally specific manner. Bilateral microinjections of lower concentrations of muscimol (10 µm) in the pre-BötC in the *in situ* preparations (*n* = 5) also rapidly suppressed phrenic inspiratory activity (**Fig. 12**).

**Figure 13.**
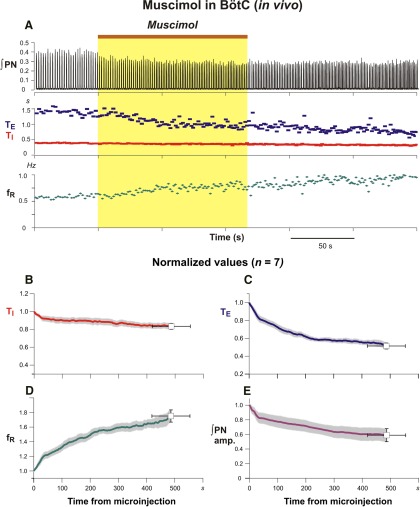
Perturbation of inspiratory activity by bilateral microinjection of muscimol in the BötC of anesthetized adult rat *in vivo.* ***A***, Muscimol (30 nl, 100 µm) slowly microinjected (brown bar at the top and yellow rectangle) in the BötC augments PN frequency due to a reduction of T_E_. ***B***–***E***, Mean time series for group data (*n* = 7) summarizing perturbations of respiratory rhythm and motor output pattern parameters. Solid colored curves are mean time and normalized parameter values and gray bands are ±1 SEM of T_I_ (***B***), T_E_ (***C***), respiratory frequency (f_R_; ***D***), and integrated inspiratory activity amplitude (***E***) computed from recordings of integrated phrenic nerve activity as in previous figures.

**Figure 14.**
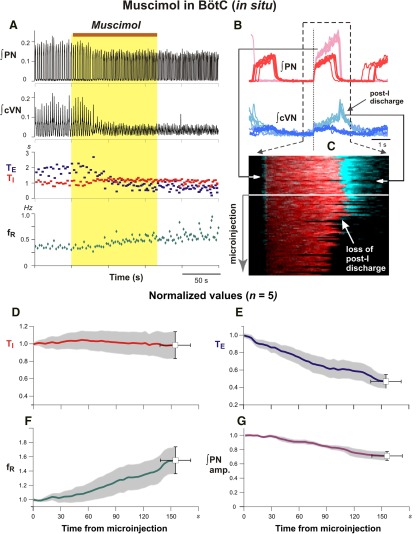
Disturbances of respiratory pattern including elimination of post-inspiratory activity by bilateral microinjection of muscimol (10 µm) in the BötC of juvenile rat perfused brainstem–spinal cord *in situ*. ***A***, Representative example of augmented ∫PN inspiratory discharge frequency and pronounced reduction of cVN integrated (∫cVN) activity amplitude due to suppression of post-I activity. Overlaid traces in ***B*** and time-series raster plot in ***C*** of consecutive respiratory cycles aligned to inspiratory onset (indicated by vertical dotted line in ***B***) illustrate perturbations of cVN post-I discharge during BötC muscimol microinjection in another experiment. ***B***, Integrated PN (red traces) and cVN (blue traces) activity are shown before (lighter red and lighter blue traces, respectively) and after (dark red and dark blue traces) muscimol microinjection. Aligned traces of ∫PN (red) and ∫cVN (blue) are shown in raster plot in ***C*** with the loss of post-I cVN discharge indicated (white arrow), which is also clearly seen from the ∫cVN traces (dark blue) above. ***D***–***G*,** Mean time series for group data (*n* = 6) summarizing perturbations of respiratory rhythm/pattern parameters. Solid colored curves are mean time and parameter values and gray bands are ±1 SEM of normalized T_I_ (***D***), T_E_ (***E***), inspiratory frequency (f_R_; ***F***), and integrated inspiratory activity amplitude (***G***) computed from recordings of integrated PN activity.

In experiments targeting the BötC *in vivo* (*n* = 7), bilateral microinjection of the same volume and concentration of muscimol progressively increased discharge frequency, due primarily to a reduction of T_E_, and reduced the amplitude of integrated phrenic inspiratory discharge (**Fig. 13**). For the averaged group *in vivo* data at 486.4 ± 67.6 s after injection onset, integrated phrenic discharge amplitude was reduced to 58.9 ± 8.9% (*p* = 0.015), inspiratory frequency increased to 175.19 ± 8.4% (*p* = 0.015), T_E_ decreased to 51.4 ± 3.3% (*p* = 0.015), and T_I_ decreased to 83.4 ± 3.3% (*p* = 0.015) of pre-injection control values (**Fig. 13*B*–*D***). Comparable perturbations were obtained with bilateral microinjections of 10µm muscimol in the *in situ* preparations (**Fig. 14**; *n* = 6). At 139 ± 20 s following the onset of the microinjections, the group averaged inspiratory frequency increased to 148.1 ± 16.88% (*p* = 0.03), T_E_ decreased to 53.1 ± 8.0% (*p* = 0.03), and T_I_ was essentially unchanged (99.0 ± 15.3% of pre-injection values, *p* = 1.0; **Fig. 14*D*–*F***). The integrated phrenic discharge amplitude was reduced to 75.4 ± 6.4% (*p* = 0.03; **Fig. 14*G***). In addition, integrated cVN post-I activity was progressively reduced and eliminated by muscimol in the BötC *in situ* (**Fig. 14*B***).

## Discussion

### Role of synaptic inhibition within and between pre-BötC and BötC for respiratory rhythm and pattern generation

For the discussion of the role of synaptic inhibition in respiratory rhythm and pattern generation, it is necessary to clarify our definitions of the terms “rhythm” and “pattern”. We define rhythm as the respiratory cycle period/frequency. Respiratory pattern is defined as all features of activity occurring within the respiratory cycle, including the presence and coordination of the different activity phases (inspiratory, post-inspiratory, late expiratory) as recorded from neuronal populations/nerve motor output activity, as well as the durations, amplitudes, and shapes of integrated activity within phases.

Brainstem respiratory networks consist of excitatory and inhibitory circuits distributed within interacting regions of the medulla and pons including the medullary pre-BötC and BötC, which are proposed to contain core circuits involved in respiratory rhythm and pattern generation. Although there is general agreement that excitatory pre-BötC circuits generate rhythmic inspiratory activity transmitted to and driving spinal and cranial inspiratory motor outputs, functional roles of inhibitory pre-BötC and BötC circuits continue to be debated (Smith et al., 2009, 2013, cf. Feldman et al., 2013; Janczewski et al., 2013; Richter and Smith, 2014). It has been postulated that phasic synaptic inhibition originating in these circuits, known to contain populations of rhythmically active glycinergic and GABAergic neurons, are critically involved in generating the three-phase pattern of respiratory neuron activity during normal breathing by shaping firing patterns of active populations of neurons, orchestrating phase transitions, and controlling which populations are inactive during each phase (Rybak et al., 2004, 2007; Smith et al., 2007, 2009,2013). Inspiratory and expiratory neurons in these regions receive phasic volleys of inhibitory post-synaptic potentials/currents as clearly established by intracellular recordings (Schmid et al., 1996; Molkov et al., 2012; Shevtsova et al., 2014; Richter and Smith, 2014), and the inhibitory neurons in the pre-BötC and BötC are proposed to interact during the respiratory cycle via mutual inhibitory connections for dynamic control of rhythm generation, although these interactions have not been definitely established experimentally.

We have further evaluated roles of the glycinergic and GABAergic synaptic inhibition within the pre-BötC and BötC by regionally disrupting/attenuating this inhibition pharmacologically with specific receptor antagonists, which has been attempted in previous studies in several species (cat, rat, rabbit) *in vivo*, most recently in adult rats with conflicting results (Janczewski et al., 2013). In the present experiments we have established that pharmacologically attenuating GABA_A_ and glycine receptor-mediated inhibition in the pre-BötC or BötC causes major site-specific perturbations of respiratory rhythm and pattern both in anesthetized, vagotomized adult rats *in vivo* and unanaesthetized, vagotomized juvenile rat perfused brainstem–spinal cord preparations *in situ*. The results obtained *in vivo* and *in situ* are congruent and confirm that:

(1) Blocking inhibition in the pre-BötC augmented inspiratory discharge frequency due to a shortening of T_E_, reduced inspiratory discharge amplitude, and eliminated post-I activity thereby disrupting the normal three-phase respiratory pattern. In some cases, rhythmic motor output was completely terminated.

(2) Blocking inhibition in the BötC reduced inspiratory discharge frequency associated with a lengthened T_E_, reduced the amplitude of inspiratory discharge, disrupted the three-phase pattern due to a loss of rhythmic post-I activity, and in some cases transiently terminated inspiratory motor output accompanying a loss of pre-BötC inspiratory activity.

Therefore, we conclude that ongoing glycinergic and GABA_A_ergic inhibition in pre-BötC and BötC circuits are fundamentally involved in dynamical regulation of respiratory rhythm generation and are required for generating the normal three-phase respiratory pattern. Our studies also confirm a fundamental role of BötC inhibitory circuits and their interactions with pre-BötC inspiratory rhythm generating circuits.

### Comparisons with results from previous targeted pharmacology studies

Our results, obtained in juvenile and adult rats, are consistent with previous studies in anesthetized, non-vagotomized adult cats (Pierrefiche et al., 1998) demonstrating that bilateral pharmacological disruption of glycinergic synaptic inhibition with strychnine in the pre-BötC reduces inspiratory discharge amplitude and markedly augments inspiratory discharge frequency or totally abolishes rhythmic phrenic nerve activity. In their studies, simultaneous block of GABA_A_ergic and glycinergic inhibition led to sustained tonic discharge on phrenic nerves, indicating disruption of rhythmic inspiratory motor output. Furthermore, results obtained in anesthetized vagotomized adult rabbits by Bongianni et al. (2010) also demonstrated that bilateral block/attenuation of glycinergic inhibition in the pre-BötC augmented inspiratory frequency and reduced inspiratory discharge amplitude. Also, blocking GABAergic inhibition with gabazine in the BötC strongly depressed inspiratory amplitude and frequency leading to apnea as we demonstrated with GABA_A_ergic and glycinergic antagonists in several cases. Moreover, in these studies in rabbits, bilateral microinjection of muscimol in the BötC potently augmented inspiratory discharge frequency and caused loss of rhythmic inspiratory activity that was replaced by tonic discharge of phrenic nerves.

The general conclusions from these previous studies in both vagotomized and vagus-intact animals, similar to our conclusions, are that ongoing neuronal GABA_A_ and glycine receptor-mediated postsynaptic inhibition in the pre-BötC and BötC have a major role in controlling the frequency and amplitude of inspiratory circuit activity during eupneic breathing *in vivo*. Other results obtained by pharmacologically blocking glycinergic inhibition with systemically applied strychnine in perfused rat and mouse brainstem–spinal cord preparations *in situ,* disturbing synaptic inhibition throughout the respiratory network, also demonstrate major disturbances of respiratory rhythm and disruption of the three-phase respiratory pattern at cellular and circuit levels (Shevtsova et al., 2011, 2014; Richter and Smith, 2014).

All of the above results and conclusions contrast with those from the recent pharmacological study by Janczewski et al. (2013) performed in anesthetized spontaneously breathing, vagus-intact or vagotomized adult rats *in vivo*. They reported that sequential bilateral microinjections of bicuculline and strychnine in the pre-BötC and then BötC via a ventral surface approach does not significantly disturb inspiratory frequency in vagotomized rats, whereas in vagus-intact rats inspiratory frequency was significantly reduced, accompanied by a prolongation of T_I_ and T_E_– perturbations that the authors attributed to only suppression of the Breuer–Hering inspiratory inhibitory reflex (BHIR), supported by their results showing that the BHIR is blocked by antagonizing synaptic inhibition in the pre-BötC. Other results presented suggested that block of inhibition in the pre-BötC and BötC does not disturb generation of laryngeal post-I activity in vagus-intact rats and thus they concluded that inhibitory circuit interactions do not participate in normal three-phase respiratory pattern generation. This result conflicts with our present and previous results (Shevtsova et al., 2011) *in situ,* where we could routinely record post-I activity on cVN or post-I neuronal activity in this unanaesthetized preparation, and found that disruption of synaptic inhibition in the pre-BötC or BötC consistently eliminated post-I activity. Another major result by Janczewski et al. (2013) was that bilateral ablation of the BötC did not significantly perturb inspiratory frequency or inspiratory–expiratory phase durations, from which they concluded that the BötC is not involved at all in generating the three-phase rhythmic respiratory pattern. This clearly contrasts with our results and those of Bongianni et al. (2010) that disrupting inhibition in the BötC can cause apnea, whereas suppressing BötC neuronal activity with muscimol augments inspiratory frequency and can lead to apneustic-like tonic discharge, similar to disrupting inhibition in the pre-BötC. Janczewski et al. (2013) presented data showing a similar augmentation of frequency leading to tonic discharge and disruption of rhythm generation with muscimol injections in the pre-BötC, which is the opposite of ours and previous results that suppressing neuronal activity with muscimol in the pre-BötC potently reduces inspiratory frequency and rapidly terminates inspiratory rhythm generation. Overall Janczewski et al. (2013) concluded that inhibition is not required for a normal eupneic breathing rhythm *in vivo* except for mediation of the BHIR in vagus-intact animals.

These major discrepancies are not readily resolved. The results obtained with these targeted pharmacological approaches depend critically on accurate site-directed delivery of the inhibitory agonists/antagonists, the ability to antagonize inhibitory postsynaptic receptors on a sufficient number of neurons by regional spread of the antagonists/agonists to ultimately perturb motor outputs, and also on regionally confining the pharmacological perturbations. In our experiments, we verified locations of injection sites histologically and we consistently obtained differential, regionally specific perturbations of inspiratory rhythm: slowing of the rhythm in all cases by inhibitory antagonists in BötC, and contrasting augmented inspiratory frequency with antagonists in pre-BötC. Our reconstructed microinjection sites appear to be identical to the sites reconstructed by Janczewski et al. (2013, their Fig. 1; compare to our **Fig. 1**). In our *in vivo* experiments using a similar ventral surface approach in adult anesthetized rats, we used identical concentrations and ejection volumes of antagonists, which we also showed, at least for gabazine, had pharmacologically effective GABA_A_ receptor antagonist-agonist interactions (**Fig. 11**). With our targeting procedures, which included mapping of characteristic neuronal activity profiles, as well as differential respiratory and blood pressure responses to local l-Glu microinjections, we also consistently obtained regionally specific perturbations with muscimol microinjections at targeted sites: reduced inspiratory frequency and apnea in the pre-BötC, but augmented inspiratory frequency in the BötC. This specificity also suggests that it was possible to confine actions of the agonists/antagonists to the targeted region. Differences in the numbers of neurons and inhibitory synapses affected by local diffusion of the antagonists might contribute, although assuming accuracy of targeting, we would expect similar perturbations because ejection volumes and antagonist concentrations used *in vivo* were identical.

### Relation to studies using selective targeting of inhibitory neurons

In a recent study using optogenetic approaches to selectively stimulate or inhibit pre-BötC glycinergic neurons by virally-transduced expression of photosensitive opsins channelrhodopsin (ChR2) or archaerhodopsin in spontaneously breathing adult mice *in vivo*, Sherman et al. (2015) found that optical stimulation of this subset of inhibitory neurons in pre-BötC *in vivo* can terminate inspiration, delay the onset of inspiration with photoactivation during the expiratory phase, and produce long-lasting apnea with prolonged photostimulation. Conversely, prolonged photoinhibition augmented the amplitude and frequency of inspiratory activity and could reverse reflex-induced apneas. This approach can provide important information on dynamic perturbations resulting from augmentation or loss of inhibitory neuron function (Abdala et al., 2015) and their results demonstrate that at least pre-BötC glycinergic neuron activity can strongly modulate ongoing inspiratory rhythm and pattern generation. The authors concluded, however, that glycinergic inhibition is not essentially involved in rhythmogenesis despite these perturbations because inspiratory rhythm persisted after photoinhibition, although as the authors discuss, it is unclear whether sufficient numbers of glycinergic neurons were optically silenced to reveal full affects of pre-BötC glycinergic neuron activity on rhythm generation.

Another important consideration for interpreting the significance of these results is that silencing pre-BötC glycinergic neurons may not be analogous to pharmacologically blocking even postsynaptic glycinergic receptors, particularly because relevant glycinergic synapses may originate from neurons outside of the pre-BötC such as BötC neurons not transduced in sufficient numbers with viral vectors targeted to the pre-BötC. These optogenetic results also cannot be readily compared with the effects of pharmacological blockade of both glycinergic and GABA_A_ergic postsynaptic inhibition. As known, pre-BötC contains inhibitory neurons with each type of transmission, as well as neurons coexpressing both GABA and glycine (Schreihofer et al., 1999; Koizumi et al., 2013), so all these neuron types would have to be optically inhibited to fully evaluate the functional role of local inhibitory circuits in the pre-BötC. The augmented inspiratory frequency that we observed with disrupting inhibition in the pre-BötC, which has also been observed by pharmacologically attenuating only glycinergic inhibition with strychnine (Pierrefiche et al., 1998) as discussed above, is a common result of both pharmacological and optical approaches. However, inspiratory amplitude was always markedly reduced, not augmented, by pharmacologically perturbing postsynaptic inhibition in the pre-BötC, suggesting that more complex inhibitory interactions in pre-BötC inspiratory rhythm generation circuits are revealed by the pharmacological approaches. Experiments were not presented by Sherman et al. (2015) on photostimulation/inhibition of glycinergic neurons in the BötC. Optical stimulation of BötC neurons transduced with ChR2, although not selectively in GABAergic and glycinergic neurons but likely including these neurons in these studies, strongly suppresses inspiratory activity in contrast to the powerful augmentation of frequency with photostimulation of pre-BötC neurons in anesthetized rats *in vivo* (Alsahafi et al., 2015). These observations are analogous to our results with local glutamate microinjections, which also do not selectively stimulate specific neuronal phenotypes in these regions.

### Normal eupneic rhythm generation in the absence of synaptic inhibition *in vivo*?

A continuing debate is whether a normal eupneic inspiratory rhythm and respiratory pattern can be generated without synaptic inhibition in the pre-BötC in the intact system where the pre-BötC interacts with the BötC and other sources of phasic/tonic synaptic inhibition. We show that disrupting pre-BötC synaptic inhibition leads either to higher frequency oscillations accompanied by a large reduction in the amplitude of pre-BötC/phrenic inspiratory activity or to tonic phrenic nerve activity in some causes, indicating that a normal eupneic inspiratory rhythm generally does not occur in the absence of synaptic inhibition. The variable persistence of inspiratory rhythmic activity in other cases, albeit with large disturbances of the inspiratory rhythm and inspiratory–expiratory pattern, indicates that either we did not sufficiently block postsynaptic inhibition in the pre-BötC with our targeted pharmacological approach in these cases, or some form of inspiratory rhythm generation occurs after disruption of GABA_A_ergic and glycinergic inhibition in the intact system. The former is a technical problem that is difficult to solve. This would require extensive local intracellular recordings to analyze endogenous postsynaptic inhibitory potentials simultaneously with the pharmacological perturbations. In our approach, we limited the microinjection volumes of the pipette solution containing inhibitory antagonists, in an attempt to achieve site-specificity of the perturbations. This may have precluded sufficient inhibitory receptor block throughout the local circuits to cause complete disruption of inspiratory rhythm generation in these cases. On the other hand, pre-BötC excitatory neurons and circuits have intrinsic rhythmogenic properties (Smith et al., 2007; St. John et al., 2009; Richter and Smith, 2014), so it is possible that some form of inspiratory rhythm generation, albeit abnormal, can persist in the absence of synaptic inhibition in the intact juvenile/adult system, as occurs in the neonatal rat/mouse pre-BötC isolated in slices *in vitro*.

Our models of excitatory and inhibitory pre-BötC circuits and their interactions with BötC inhibitory neurons in the intact system incorporate intrinsic rhythmogenic properties in the excitatory pre-BötC population (Rybak et al., 2004, 2007; Smith et al., 2007; Rubin et al., 2009). These models indicate that depending on the initial excitation state of the excitatory kernel neurons, the endpoint after complete block of postsynaptic inhibition can be either tonic activity of pre-BötC neurons at high levels of excitation or rhythmic activity at lower excitation levels. It is clear that to answer the question of whether any form of inspiratory rhythm generation can occur, intracellular recordings will have to be used to monitor the level of membrane potential and activity patterns during and after local block of postsynaptic inhibition.

### Disorganization of the three-phase respiratory pattern after disrupting postsynaptic inhibition

The three-phase organization of the respiratory pattern at least *in situ* was disrupted accompanying the large disturbances of inspiratory rhythm by blocking inhibition in the pre-BötC or BötC. This confirms that the normal eupneic breathing pattern with appropriately coordinated spinal and cranial motoneuron activity relies on synaptic inhibition in BötC and pre-BötC circuits. These inhibitory circuits normally operate with pontine and other excitatory inputs (Rybak et al., 2004; Smith et al., 2007; Dutschmann and Dick, 2012). The pontine Kölliker–Fuse nucleus projects heavily to both the pre-BötC and BötC affecting their inhibitory interactions. Specifically, pontine input is required to generate post-I activity (Rybak et al., 2004; Smith et al., 2007; Dutschmann and Dick, 2012) so the observed loss of this activity in part reflects disruption of the balance of excitatory and inhibitory interactions. Recent experiments in a transected perfused rat brainstem preparation have confirmed the critical role of the pons in three-phase respiratory pattern generation (Jones and Dutschmann, 2016). Previously it has been demonstrated that blocking glycinergic inhibition systemically causes a shift of post-I neuron activity into the inspiratory phase, due to phasic excitation of post-I neurons that is normally shunted by inspiratory phase inhibition (Richter and Smith, 2014) and this shift causes abnormal glottal constriction during inspiration (Dutschmann and Paton, 2002). This also implies that the shifted post-I inhibitory neurons would provide abnormally timed inhibition during the inspiratory phase, possibly including to inspiratory neurons in bulbospinal circuits downstream from the BötC and pre-BötC, which could contribute to the large reductions of inspiratory amplitude and attenuation of PN ramping inspiratory discharge observed after disrupting inhibition. The loss of the three-phase pattern is predicted from models (Rybak et al., 2004, 2007; Smith et al., 2007; Shevtsova et al., 2011, 2014) after inhibitory block in the BötC, where post-I inhibitory neuronal activity is presumed generated. Our results that the three-phase pattern is also disrupted after blocking inhibition in the pre-BötC may reflect the known presence of post-I neurons in the pre-BötC (Schwarzacher et al., 1995; Alheid and McCrimmon, 2008), and shows that the pre-BötC also plays a role in three-phase pattern generation, conceivably by either interactions with the BötC and/or by local inhibitory circuit interactions, which need to be delineated.

### Role of BötC circuits and synaptic inhibition in respiratory pattern generation

A fundamental role for BötC neurons in respiratory pattern generation has been questioned by Janczewski et al. (2013) as noted above. Their conclusion that this region plays no role is incompatible with the present and previous results showing that augmenting BötC neuron activity (by glutamate microinjections) suppresses pre-BötC inspiratory activity, whereas suppressing BötC activity (by muscimol) augments pre-BötC inspiratory rhythm. The BötC is known to contain major populations of active excitatory and inhibitory expiratory post-I and aug-E neurons, and this region is critical for generating post-I activity (Burke et al., 2010). Excitatory BötC neurons are thought to be a major source of expiratory activity in the respiratory network including for generation of post-I premotoneuron activity. BötC glycinergic and GABAergic expiratory interneurons are proposed to provide widely distributed network synaptic inhibition during expiration (Jiang and Lipski, 1990; Tian et al., 1999a,b; Ezure et al., 2003a,b). These postulated inhibitory connections include to pre-BötC excitatory and inhibitory inspiratory neurons to provide phasic synaptic inhibition orchestrating rhythmic alternation between expiratory and inspiratory activity in the network. Mutual inhibitory synaptic interactions between BötC inhibitory (post-I and aug-E) expiratory neurons and inhibitory inspiratory (e.g., early-I) pre-BötC neurons have been proposed to coordinate generation of the three-phase pattern of neuronal activity during eupneic breathing (Rybak et al., 2004, 2007; Smith et al., 2007; Rubin et al., 2009; Shevtsova et al., 2011, 2014; Richter and Smith, 2014). Based on these proposed interactions, augmenting BötC activity or disrupting postsynaptic inhibition within the BötC should suppress rhythmic activity in the pre-BötC, assuming in each case that BötC neurons shift from phasic to tonic activity. Correspondingly, suppressing BötC activity should augment inspiratory discharge frequency, and depending on the level of ongoing pre-BötC excitation, this can lead to tonic inspiratory activity. Both of the above are features of the present experimental results.

We note that the projections and postsynaptic targets of BötC and pre-BötC inhibitory interneurons, particularly mutual inhibitory connections between these neurons, have not been mapped structurally. Establishing these connections remains an important problem. Nevertheless, our results are consistent with mutual interactions and a fundamental role of the BötC and its expiratory inhibitory neurons in three-phase respiratory pattern generation and control of inspiratory rhythm.

## Synthesis

The decision was a result of the Reviewing Editor Douglas Bayliss and the peer reviewers coming together and discussing their recommendations until a consensus was reached. A fact-based synthesis statement explaining their decision and outlining what is needed to prepare a revision is listed below. The following reviewers agreed to reveal their identity: Mathias Dutschmann, Muriel Thoby-Brisson

Two reviewers have assessed this report, and they concur that the paper provides fundamental and essential work regarding the role of fast synaptic inhibition in respiratory rhythm and pattern formation. Although generally pleased with the study, they raise important concerns regarding methodology (e.g., regarding injection volumes, durations, locations; measurements of frequency in tonically-active preparations, etc.) and data presentation (e.g., figures may show extreme examples, etc.). In addition, a number of points should be addressed regarding inconsistent terminology, and interpretation of the data and conclusions. The detailed comments from both reviewers are provided herewith.

Reviewer #1

The research article "Perturbations of Respiratory Rhythm and Pattern by Disrupting Synaptic Inhibition within Pre-Bötzinger and Bötzinger Complexes" investigates the contribution of fast synaptic neurotransmission in 2 critical areas of the medullary respiratory networks. The data are well presented and analyzed and are of importance to understand the fundamental processes of respiratory rhythm and pattern generation. While, the experimental protocols and data are basically valid however, I have some queries regarding the interpretation of the data.

Major point:

The effect of drug injections in down or upstream elements in particular the pons needs to be addressed more thoroughly. For instance the pontine Kölliker-Fuse projects heavily to both Pre-Bötzinger and Bötzinger complex and thus some of the observed effects could be due to changes of ponto-medullary synaptic interaction - please discuss these possibilities particular in the context of respiratory pattern formation - I am not aware of data showing that normal eupneic pattern formation occurs in absence of the pons or critical sensory feedback loops (e.g lung stretch receptors)

Minor

Results

First line: I puzzled you get such strong respiratory modulation after injection of just 5nl glutamate? - Injection volume for gabazine/strychine were 110 nl - please comment on this substantial mismatch of drug volumes injected?

L-Glu microinjections into BötC (n = 10) always immediately caused transient apnea (from 5.4 to 21 s; 10.1 {plus minus} 4.03 s) - I guess it would be more apporiate to state that glutamate prolonged the respiratory interval since this is what you have analyzed.

BötC injections:

A previous publication (Burke PG, Abbott SB, McMullan S, Goodchild AK, Pilowsky PM. Somatostatin selectively ablates post-inspiratory activity after injection into the Bötzinger complex. Neuroscience. 2010 May 5;167(2):528-39. doi:10.1016/j.neuroscience.2010.01.065) reported that injection of the inhibitory neuropeptide somatostatin into the BötC triggered apneusis in a in vivo preparation - any idea why your injections did not to trigger the expected apneusis but instead a shortening of Ti? - see figure 13

-Please comment on this discrepancy between your study and the previous study-.

Later you discuss 'In these studies, simultaneous block of GABAAergic and glycinergic inhibition lead to tonic (apneustic-like) discharge on phrenic nerves, indicating disruption of rhythm generation'. Is apneusis considered as disruption of rhythm generation by the authors? Apneusis is disruption of the pattern and disturbance of the rhythm but not a disruption in my view - otherwise the pontine respiratory groups should be considered as primary rhythm generators as well (block of pontine activity in vagotomized animals/preparations consistently produce Apneusis and thus according to your definition should be then a critical area for respiratory rhythm generation? - please refine and expand this point in the discussion....

Figures

I notice that in some experiments respiratory activity appears to recover rather quickly - 2 min? - after gabazine/strychnine microinjection - such quick recovery from drug effects after local injections are rare - other traces of original recordings imply longer-lasting effects? In the context of your very well localized microinjection sites I find this a bit strange? Please comment.

Discussion:

It would be great to start your discussion with a definition what is considered as respiratory rhythm and what is considered as respiratory pattern - I feel that this terminology is used in quite different levels and so sometimes your interpretation of your own results and these of other might be confusing.

Reviewer #2

The present paper submitted to eNeuro investigates the consequences on breathing activity/pattern of perturbing synaptic inhibition in two structures involved in respiratory rhythmogenesis, namely the preBötzinger complex (preBötC) and the Bötzinger complex (BötC). The authors performed their experiments using two different models: anesthetized adult rats in vivo and perfused in situ brainstem-spinal cord preparations obtained from juvenile rats. They injected either Gabazine+strychnine to antagonise GABA and glycine receptors or Muscimol to activate GABA receptors in the two structures of interest (independently) and measured the effects on respiratory frequency, inspiratory time (Ti), expiratory time (Te) and on the three phase respiratory pattern. Several anatomical and functional controls were performed to ensure targeting the appropriate networks. They found that disrupting inhibition in both the preBötC and the BötC leads to site-specific perturbation of the rhythm and the pattern. They conclude that inhibition plays an important role in respiratory rhythmogenesis and is required for normal respiratory pattern generation.

My first concern is about the injections and measurements: on every figure the injection duration is different. What determines this duration? Is it the total volume to inject? Why is it so variable from one preparation to the other? Then, despite the explanations in the method section (pages 13-14) it is difficult to understand how mean values for changes could be measured as the values cannot be obtained at the same comparable time; even more striking when we consider that the response time course is variable among experiments.

Linked to this it is unclear how frequency could be measured for in situ preparations injected with Gabazine and Strychnine (Figure5). Indeed it is written that some preparations ended up with a tonic activity. The authors should clarify at what time they measured the different parameters, did they exclude tonic preparations?.....

My second concern is on the samples used for the figures that seem to be for some of them particular extreme cases. For example Fig5A (seem to be 1 over 6 experiments), Fig6A (2 over 6 experiments), Fig7A (1 experiment). So it is unclear whether these recordings are representative of the mean effect or the more impressive ones.

I am wondering how correct it is to interpret the same way experiments in which inhibition is blocked with experiments in which the activity of a network is blocked (by enhancing GABAergic inhibition). Indeed in the first set of experiments inhibition is blocked wherever it comes from, while in the second set of experiments the entire activity of the network is affected by muscimol and therefore includes blockade of both excitatory and inhibitory this network sends to its target.

The authors should discuss the fact that by blocking inhibition in the preBötC, the BötC or by inhibiting the BötC they always observed the loss of post-inhibitory discharge and a decrease in inspiratory discharge amplitude (sum in page 21). It is a good argument in favor of an important role for inhibition in controlling the respiratory pattern but it is complicated to link this with the different respiratory phases controlled by each neuronal group.

The authors should explain why they used two different preparations. What is (are) the advantage(s) of having done the experiments in vivo and in situ?

It is obvious that the present study has been done to be confronted with Janczewski 2013 paper that proposes opposite conclusions. In this context, because the differences between the two studies are not so clear and that one possible explanation for reaching different conclusions could be the quality of the injections wouldn't it be useful to illustrate on one map the location of all the injections performed in the different experiments. This would strengthen the fact that the injections were all performed in the expected spot indeed.

Although it is important to discuss the discrepancy between the two studies I was more interested in the mechanistic hypothesis explaining the observed effects. So I would address hypothesis for the disorganization of the pattern and the role of BötC and preBötC in respiratory rhythmogenesis first in the discussion (but this is my personal feeling).

Minor comments:

-Figure 9: the way it is presented, the traces before and after treatment are not so easy to distinguish. In the legend it is referred to upper traces and lower traces. Wouldn't this be indeed the best way to present the data?

-Figure 3 legend: post-I is cited in the text but not present in the figure.

-Introduction: I am surprised that the authors are not citing the RTN/pFRG as an important group involved in respiratory rhythmogenesis.

-Introduction, line 12: not all excitatory neurons have intrinsic bursting properties. Please rephrase.

-Introduction, line 17: the work by Morgado-Valle on glycinergic pacemaker (2010) should also be cited here.

-Results, page 15, end of the first para: are the given values corresponding to the duration of the apnea? Is it the range and then the mean value? Please clarify.
